# Fostering Well-Being in the Elderly: Translating Theories on Positive Aging to Practical Approaches

**DOI:** 10.3389/fmed.2021.517226

**Published:** 2021-04-09

**Authors:** Liora Bar-Tur

**Affiliations:** MA Program in Gerontological Clinical Psychology, Faculty of Social & Community Sciences, Ruppin Academic Center, Hadera, Israel

**Keywords:** mental health, well—being, positive psychology, successful aging, positive health

## Abstract

This article overviews positive aging concepts and strategies to enhance well-being in the elderly and then presents a translation of theories on positive aging to practical approaches for Positive Aging. Drawing upon positive psychology and positive aging research and tools, this program is designed to help older adults improve their well-being by acquiring skills and strategies to cope with present and future challenges. The Mental Fitness Program for Positive Aging (MFPPA) can enhance seniors' quality of life by increasing their vital involvement and active engagement in life. This model is most appropriate for community dwelling individuals. It can easily be conducted in wide range of adult education programs in community centers, sheltered homes, and primary care clinics. It can also be conducted through online psychoeducational training.

## Introduction

The world is facing a demographic revolution; life expectancy has risen by more than two decades since 1950 and the population has grown considerably. According to the World Health Organization (1), the number of people over 60 in almost every country is growing faster than any other age group. This reflects a combination of influences including increased longevity, declining fertility, and the aging of the “baby boom” generation. These increases in longevity and the quality of life of older adults constitute a challenge to mental health professionals working to help the growing older population not only to live longer and healthier but also better and happier. How to age successfully and embrace well-being is the focus of much concern.

Traditionally, at least in Western societies, the elderly were viewed as “irrelevant” and a financial drain on society. Recent studies have shown that many older adults are relatively healthy, active, and independent, and have many more resources for aging successfully and maintaining high levels of well-being. A growing number of older adults are resilient, socially engaged, and involved in their families and communities (2–5).

Growing old in the twenty-first century is still a challenge and entails high risk, especially for the “old-old” and the “oldest-old.” Thus, aging successfully depends to a large extent on coping effectively with age-related life events.

This article describes strategies and an intervention to enhance older adults' positive functioning and well-being (6, 7) based on theories of positive aging and positive psychology research and interventions. Positive psychology, in theory and practice, centers on the notions of well-being and optimal functioning. Research on positive aging deals with the comparative psychological well-being of older adults (8), their mental and physical health, concepts associated with aging successfully (9–12), and how psychological well-being can contribute to better health (13–15). Rather than emphasizing dysfunction and psychopathology, the emphasis is on ways to flourish and increase functioning. Positive psychology studies have put forward techniques to deal more effectively with key transitions over the course of the lifespan (14–17). In this sense, research on positive psychology and its principles can be harnessed to support positive aging.

Many active seniors do volunteer work or have part-time jobs where they can still contribute to family and community welfare (5). However, although the elderly continues to make a contribution to their society, there are still extensive disparities in the way people experience aging and its many physical and cognitive impairments (6, 18). This makes it imperative to better understand what constitutes successful aging and in particular how the lens of positive psychology can support and foster mental and physical health.

## Positive Aging: A Challenge for Older Adults' Mental Health

Positive aging is a multidimensional concept that combines various characteristics of aging well such as optimal, successful, productive, and healthy aging. Positive aging consists of five independent factors: health, cognition, activity, affect, and physical fitness. It is described in practice by a broad set of biopsychosocial factors and is assessed through both objective and subjective indicators. The basic notion is that at any age, including the old and very old, people are to some extent in charge of and responsible for their own quality of life (2). They can enjoy positive well-being and experience “successful aging.” Various models of successful aging (8, 9, 19–21) have explored the components and dimensions of well-being and positive function in older adults.

Ryff's integrated model (originally called “successful aging” and later psychological well-being), was first presented by Ryff (19) and Ryff and Essex (22) and later by Ryff and Singer (8, 23) and Ryff (20). It incorporates lifespan developmental theories, clinical theories of personal growth, and mental health perspectives. It comprises six dimensions of positive functioning: self-acceptance, positive relations with others, autonomy, environmental mastery, purpose in life, and personal growth.

Research has revealed that these dimensions of well-being and healthy mental functioning are shaped by socio-demographic characteristics (e.g., age, gender, socio-demographic status, ethnicity, and culture), as well as by individual experiences, including both unexpected life stresses and planned, normative transitions (22–25). Hedonic well-being generally shows gains with age; that is, older adults tend to show increments in positive affect and decrements in negative affect, at least until very old age. In contrast, eudemonic well-being, reflecting a sense of purpose, is more inclined to reflect decrements in the later years, especially in terms of assessment of purpose in life and personal growth (8). In-depth measures across multiple domains as well as longitudinal follow-ups have been conducted since Ryff (19) first introduced the successful aging model. One of the most prominent studies is MIDUS (Midlife in the US, www.midus.wisc.edu).

The MIDUS findings documented that multiple psychosocial factors such as purpose in life, social relationships, mastery, and prosocial behaviors such as volunteering predict better self-rated health, less disability, healthier profiles of biological risk, greater well-being, and better cognitive function in aging adults, even in the context of disability and chronic illness (26). Other findings suggest that resilience in the face of age-related challenges may involve not only a better quality of life but also a reduced risk of future disability and death.

Growing neuroscientific evidence linking psychological well-being to physical health, and biological regulation shows that qualities such as purposeful engagement, self-realization and growth, and enlightened self-regard influence how long and how well people live. Some studies have linked illnesses and disabilities to different aspects of well-being. Epidemiological studies have documented the protective influence of well-being (especially purpose in life) in reducing later life ill-health and cognitive impairment (25). Even positive emotional experiences such as enjoyment can help reduce anxiety and thereby indirectly decrease cortisol production (26). Epidemiological studies have suggested that the degree of purpose in life predicts reduced risk for numerous diseases (Alzheimer's disease, stroke, myocardial infarction), and a proliferation of studies have begun to probe the underlying biological mechanisms of neuroendocrine regulation, inflammatory processes, gene expression, glycemic control, and cardiovascular risk on aging. Many more studies have linked phenomenological indicators of well-being to reduced amygdala activation in response to negative stimuli, sustained activity in the ventral striatum and dorsolateral cortex when viewing positive stimuli, and increased insular cortex volume [for more details see Ryff (25)].

All these analyses in the fields of phenomenology, biology, genetics, and neuroscience contribute to accounting for adaptive human functioning (20, 25). Positive aging studies have found that older adults can optimize their aging experience (20, 25, 27). They can maintain and implement preventive health behaviors, act on resources available to them to cope with age-related decline, and increase their well-being (7, 28). Thus, the focus should be on wellness, not illness in psychological research and in interventions for the older population.

## Interventions

The broad scope of studies on psychological well-being and its complex links to mind-body and socio-cultural factors have fostered important new directions in applications and education. Interventions to enhance and improve older adults' well-being are on the rise. These advances have been accompanied by preventive interventions outside the clinic (25, 29, 30).

Numerous intervention studies with older adults in the community, clinics, and in nursing homes have reported noticeable improvements in their psychological well-being (30–36), subjective happiness (36–39), and life satisfaction (30, 40, 41). These changes in well-being are accompanied by improvements in the quality of sleep (30, 33), better working memory (32), decreased anxiety (36), higher levels of overall mindfulness (42), and improvements in self-reported feelings of depression and depressive symptoms (30, 34–36, 38, 39, 42). Although these well-being interventions suggest that the older population's quality of life can be improved, studies with more rigorous designs and extended follow-up measurements are needed to consolidate these positive findings.

The major purpose of this wide range of interventions is to reach as many older adults as possible, especially those who avoid seeking psychological support because of their negative beliefs and attitudes toward psychotherapy or mental health professionals. Older adults should be provided with the necessary support to maintain a good balance between their decreased physical ability and increased transcendence, which can be significantly enhanced by tapping personal, environmental, and social resources. This equilibrium includes physical and mental health at the micro level (personal), social well-being, and spirituality/transcendence (process) at the meso level, while living in a favorable and appropriate environment (43). Many older adults constitute a resourceful group that can contribute actively to society, realize their own potential, cope with normal stressors of life, and contribute to their community productively and fruitfully. They implement what is called proactive coping, which is defined as “an effort to build up general resources that facilitate promotion toward challenging goals and personal growth” [(44), p. 349].

The challenge facing professionals working with older adults is to convey information about positive aging and suggest strategies to increase well-being by teaching new skills for positive functioning. This can be achieved by recruiting and training staff members in health centers, primary care, sheltered homes, and other community centers and services for older adults who can help incorporate strategies and tools for optimal functioning in their organizations and communities.

## Strategies To Enhance Positive Aging

### Comprehensive Assessment of the Older Person's Positive Health in Primary Care Services and Mental Health Clinics

Many medical professionals, and specialists in particular, tend to overlook the constitutive unity of the person as a whole in the context of this individual's physical, social, and mental surroundings. Any comprehensive evaluation in mental health clinics, hospitals, and primary care should include an in-depth examination of the person's life, beyond his or her cognitive and physical condition. This means including information as to the person's social, cultural, historical, and spiritual background to assess the individual's positive health. This information can be obtained prior to the consultation by the secretarial staff, interns, or students and should be transmitted to the doctor. This is particularly true for depression, which constitutes the most common emotional disorder in older adults. Milder forms of depression such as dysthymia are known to affect 20–30% of all older adults (45). Nevertheless, older people are rarely offered psychological interventions (46). Many older patients who suffer from depression remain undetected in primary care. Studies show that somatization is one of the most important single problems associated with this missed diagnosis (47). The risk of suicide among older men is the highest mostly as a result of chronic physical illnesses and disability (48). Research has shown, however, that 20% of these individuals had consulted a physician that same day, and 40% in the same week that they committed suicide (49, 50). Medical staff are often “gate- keepers” who meet older adults who may be at risk for psychological and social problems. Awareness of the inseparable relationships between physical and mental health can lead to comprehensive assessment of the older person's positive health. This is necessary if we wish to prevent and detect mental health issues as well as to enhance optimal well-being of older adults.

### Promoting Positive Health

The objective of positive health programs is to encourage seniors to be more cognizant of their own resources and their strong health points to cultivate their rich physical, cognitive, and social qualities. These programs should be conducted as wellness programs offered by primary care clinics or health clubs in the community that are designed to encourage better health through changes in diet, cutting down on cigarettes and alcohol, getting more exercise, and learning ways to manage stress and increase relaxation. Taking these steps toward maintaining a healthier lifestyle can reduce the impact of disease (51). These guidelines may also have a positive effect on cognitive decline and mood swings. Healthcare professionals may be engaged in the implementation of these interventions and reach out to older adults in their community to encourage them to join health promotion programs and engage in interventions that enhance positive aging. Primary care clinics can also offer a wide range of preventive interventions such as fall prevention and disability interventions for the frail and elderly (52, 53).

### Promoting Optimal Aging

A systematic review and analysis of the effectiveness of 69 psychosocial interventions to promote the mental health of older adults (54) revealed that skill training interventions with educational and/or behavioral components had a significant effect on positive mental health outcomes. Promoting optimal aging can be achieved by implementing special training programs for older adults in the community including in leisure clubs, country clubs, retirement villages, sheltered homes, and homes for the aged. They can also be conducted through online psychoeducational training. These training programs can include, for example, the ABC model (Activating events-adversities, Beliefs-Consequences) of optimal aging, which is grounded in the notions of unconditional self-acceptance and the frustrations linked to the inevitable consequences of aging (55). Alternatively, the six “keys” for positive functioning can give the elderly more meaning and purpose (8, 13, 20) where seniors are encouraged to acquire a more positive attitude toward one's own self and the past including improving interactions with significant others. Seniors can work on their autonomy, learn to have a voice, and reinforce their capabilities to make decisions affecting their lives. Mastering one's environment by being able to handle daily life and create surroundings adapted to one's needs is another key. One way of having renewed purpose in life is to reassess the present and the past, or engaging in social activities. Senior volunteers, for example, were found to be happier, calmer, more content, more fulfilled, and more vital (56). Older adults who are able to capitalize on these five keys are more likely to experience the sixth key of personal growth and are more open to experiences and capable of greater further development.

### Positive Assessment of the Older Person's Strengths and Reserve Capacities

Practitioners and therapists working with older clients should move away from the traditional medical assessment model. Instead of focusing on the older person's weaknesses (a frail body, depressive moods, or weak community relationships), professionals should assess and activate clients' strengths and reserve capacities, and the strengths of the key primary environment (family, friends, close associates, etc.) and secondary groups (large-scale organizations, communities, cultural groups, etc.) to reach personal goals and prevent predictable problems (57, 58). Thus, rather than asking “What is wrong?” (the typical question asked when visiting the physician), mental health professionals should aim to determine “What is good about me? What is still working? What is my reserve capacity?” (6, 59). Positive assessments can be combined with the physical assessments and should be given to the older person as part of the overall positive health assessment.

### Positive Psychology Interventions to Cope With Loneliness and Depression

Loneliness is a significant risk factor in the emergence of mental and physical health problems (60). When meaningful social connections are perceived as severed or unavailable, loneliness can have deleterious effects on cognition and behavior (61). Interventions in healthy older adults such as physical or dietary changes and enhancing social and cognitive engagement can help diminish the impact of loneliness on the aging process and the emergence of psychiatric disorders (62).

Older people with depressive disorders can receive additional cognitive behavioral therapy, which is an effective treatment for depression and anxiety (46, 63, 64). In a systematic review (65) examining the utility of 34 loneliness alleviation interventions among older persons, the findings suggested that loneliness can be reduced by using educational interventions focused on social network maintenance and enhancement. These interventions can be implemented directly in face-to-face meetings, group psychoeducation encounters, or through tele-help online interventions. Today, mental health clinics are rapidly converting from face-to-face modes of care to virtual ones as a result of the COVID-19 pandemic. Since the beginning of the pandemic, many older adults have opted to stay at home and avoid attending adult day centers and other community programs. This underscores the importance of establishing a system of remote mental care, comparable to what is being implemented for physical healthcare (66–68).

The telephone and video substitutes for in-person meetings pose an enormous challenge but also an opportunity to reach out to many older adults who otherwise would not receive emotional support. A support system could be established, for example, by initiating substantive online conversations to ascertain that their emotional and mental needs are being met and to encourage them to ask for help, and to share. This would contribute to reducing the loneliness and isolation that accompany long-term lockdowns. Studies have shown that writing down three good things or blessings every day, as well as engaging in gratitude and savoring techniques, can contribute to positive states and reduce feelings of depression related to ill-being, negative thoughts, and loss of a sense of meaning (69–71).

### Changing Professionals' Attitude Toward Positive Aging in Medical Staff, Old-Age Home Staff, and Other Professionals Working With the Older Population

Positive psychology programs are likely to have more impact when those running them are themselves positive and healthy. This includes a better understanding of the aging process in the medical profession, which may still see aging as purely negative (68, 72, 73). There should be a concerted effort to expand the basket of psychological health services offered to the elderly, which would alleviate the strain on the health system through prevention, detection, and rapid responses (74).

### Enhancing a Sense of Community and Connectedness

Fostering dialogue and a sense of community can help seniors increase their social and emotional support, find ways to preserve and enrich family, friends, and community ties, and take advantage of neighborhood, community, and social activities to cope with anxiety and feelings of isolation. One major step involves proactive interactions to contact seniors without a strong social network by providing regular ways to “check in” by including them in social events and structured activities for older adults, as well as psychoeducation and support. Internet skills are a plus. Seniors can be taught to search for content of interest to their age group, read local newspapers, get medical information, find out about events likely to appeal to them, and ways to socialize through emails, WhatsApp, Facebook, etc. Studies have shown that being computer savvy reduces isolation and reinforces a sense of autonomy (75).

Overall, positive psychology interventions can be applied in health settings, but they should also be conducted in settings already designed for positive interactions, such as senior citizens' clubs and retirement communities. Modern media, including radio, TV, and the internet, are important ways to connect and reach out to the elderly. By teaching seniors fundamental techniques that can lead to greater optimism, enhanced positive thinking, and ways to define objectives that lead to higher involvement and meaningful participation in the community, older adults can benefit from an improved quality of life. Systematic reviews of interventions designed to enhance the well-being of older adults indicate that group-based interventions and interventions including social components have a very positive effect on participants' mental health (54). This suggests that group-based interventions should be prioritized.

## The Mental Fitness Program for Positive Aging

The Mental Fitness Program for Positive Aging is designed as a psychological journey with 12 stations, each addressing a different topic. The program can be applied in a group setting or individually through personal coaching or counseling. The program includes a “personal map, compass, sail and oars” to help senior citizens plan how they want to live their lives in the near future and what they wish to experience along the way (55). These combined tools provide participants with a way of developing positive attitudes toward aging by encouraging them to review their past accomplishments and their current personal resources and strengths. Various positive psychology strategies are introduced in each session, such as exploring personal strengths and practicing new ways to use them, learning how to invest in significant relationships, visualizing one's best self, keeping a gratitude diary or a list of good things occurring during the day, seeking out activities that create flow, practicing mindfulness and acts of kindness, or accessing stories from their own lives to hone their sense of hope (6, 17, 76). Homework assignments for practicing these strategies are included together with other assignments relevant to each session's topic and assignments that are addressed to maintaining healthy lifestyle.

**Table:** 

Phase 1	The journey begins with an introductory lecture by the group facilitator, who reviews the core and the latest studies in positive psychology and positive aging. Then, attendees decide whether they want to continue as participants. Registering for the group can be viewed both as a proactive step toward change and an indicator of willingness and commitment to attend most of the sessions and execute the homework assignments.
Phase 2	The second phase addresses the issue of identity and the aging self. The focal task is to increase self-acceptance and positive self-image. Participants are guided in posing the following questions to themselves: “Who am I?” and “Who am I at this particular phase of my life?” Each participant is asked to write a personal introduction card in the form of several bullet points, by way of response to the above questions.
	The purpose of this exercise is to discuss individual identity and activities toward reinforcing self-acceptance and positive self-esteem. Discussion questions are: “What does it mean to you to be 70+ years old? How do you feel as someone who is retired? What is your role as a grandmother or grandfather?” and others. By discussing this “business card” the third ager can evaluate his or her life satisfaction, mental and emotional engagements in life, and personal attitudes. The bulleted identity points written on the card raise important reflective questions such as “What are my roles and meaningful engagements in life? Do I present myself in a positive or negative light? Do I emphasize my achievements or my shortcomings? Do I dwell on the past and my losses or emphasize the positive aspects of the present?” By analyzing the “business cards,” the group and facilitator can learn a great deal about the participants' well-being.
	The facilitator then helps the participants highlight their positive experiences and strengths and to shift from thinking about what they can no longer do to what they can achieve in the present. Participants are asked to examine their reserve capacities.
Phase 3	The Mental Fitness Program encourages senior citizens to examine their rich pasts as well. Reviewing past experiences and applying understanding and acceptance to disappointments and failures allows the participants to “draw” a road map of their talents and skills and the domains in which self-efficacy has been cultivated. The focus of the discussion is on achievements and accomplishments in life rather than dwelling on negative experiences, which are nevertheless recognized. Each person is given a picture of a boat sailing in the ocean. The guiding questions are: “Where do I come from and where do I want to sail to in the near future?” Participants should provide a short description of their past experiences with a focus on significant events and milestones in which they can evaluate their strengths and personal resources. When discussing negative or traumatic experiences, participants should reflect on how those were overcome and what capabilities and internal strengths helped them cope and adapt.
	Maintaining positive self-esteem is often associated with the quality of interpersonal relations; thus, the first three sessions also focus on mapping and assessing relations with meaningful others such as family members, friends, colleagues, or others. Positive self-esteem also relates to the senses of autonomy, environmental mastery, self-realization, and personal growth, with the latter serving to enhance the pursuit of life's goals. Senior citizens who were fully engaged in work or social roles, as well as widows and widowers who were assuming the role of care-giver, may need guidance in finding suitable alternative contexts for gaining a sense of control and purpose in their lives.
	In gaining a sense of direction and a realistic view of identity and self-image, the participants are enabled to discuss their priorities, and personal and independent decisions, before planning and setting goals to maintain or increase their well-being. The program teaches the participants to identify positive, realistic goals and to use the adaptive mechanisms of selection, optimization, and compensation (SOC) (9) to promote continued maximization of their chosen activities in life, especially as they grow older and their resources decline (77).
Phase 4	In this phase participants focus on their purpose in life through the process of goal attainment. Setting clear, realistic, measurable, and significant goals and managing time and learning how to spend time effectively become increasingly important elements as people age and find they have more free time and fewer commitments to family, work, and other obligations (78).
	Thereafter, participants identify and increase their health-promoting behaviors, to cope with the adversities encountered in growing old. Since unrealistic expectations, beliefs, and attitudes regarding old age affect well-being and impede goal attainment, they learn to identify and replace irrational beliefs and attitudes with rational, more realistic ones (28). Assisted by the group, the participants individually and collectively seek and create emotional and social support. Participants are encouraged to form a social network using the internet or phone communication, which is activated between the meetings for staying in touch, receiving support, and sharing experiences, thoughts, and feelings experienced during the week.

The Mental Fitness Program can also be an effective intervention tool to assess senior citizens' main areas of personal difficulty. It helps ascertain which components to positive well-being would benefit from enhancement, such as low self-esteem, unsatisfying relations with others, lack of emotional or social support, or difficulties in making decisions and taking the steps to fulfill personal needs. It may also indicate the phase in life where changes and losses require that personal goals be revised to promote emotional and psychological engagement and gratification. For seniors who suffer from emotional problems or mental disorders, positive psychology interventions can be integrated into general treatment, such as positive psychotherapy, CBT-REBT, or IPT (Interpersonal Psychotherapy) for depression (28, 79). These interventions can be successful for helping professionals who are themselves optimistic and in good health in facilitating seniors. However, many professionals still need to overcome their own ageist attitudes and phobias if they are to effectively assist what will be an increasingly larger proportion of their clientele (72, 73, 80). Professionals should also encourage clients to maintain a healthy lifestyle and are encouraged to engage in strategic advocacy for extended medical insurance for mental health services, mainly in the areas of prevention, screening, and early intervention.

**Implementation:** This model is most appropriate for community-dwelling individuals with a high level of functioning. It can easily be conducted in a wide range of adult education programs in group settings, in community centers and health clubs. Seniors can be recruited through seniors' communities online (Facebook). It can be offered to members of health-care insurance companies as part of programs suggested to promote health aging. It can also be implemented online as is currently the case via Zoom. The Mental Fitness Program has been applied in Israel in community centers, sheltered homes, and retirement programs and has proved to be very effective, as shown by the excellent feedback provided by the participants. Mental health professionals were recruited to lead the group sessions after attending a full-day training course on how to conduct the program. The instructions for the mental fitness program are clear, and social work or psychology students can also be recruited to lead the group sessions.

**Limitations:** The mental fitness program is one of many interventions suggested to enhance the well-being of older adults. Its advantage is that it is a comprehensive program of positive health combining the physical, cognitive, social, and emotional aspects of positive aging. The limitations of this model have to do with the fact that it is most appropriate for community-dwelling individuals with a high level of functioning as compared to individuals who have experienced cognitive decline. Another limitation is that to date, the model suggested has not been methodologically evaluated. An evaluation of this model should be performed and should be tested methodologically. The decision as to the type, frequency, and length of any strategy is, however, not easy to estimate. It depends on the institutions, and the older adults' current capacity, vulnerability, and subsequent adherence to the intervention (52, 62).

Systemic reviews of interventions designed to enhance the well-being of older adults indicate that group-based interventions and interventions including social elements have a very positive effect on participants' mental health (54). This suggests that group-based interventions such as the Mental Fitness Model for Positive Aging can be effectively prioritized.

We hope that the Mental Fitness Program will be evaluated shortly and will be approved as an effective intervention in promoting positive well-being of older adults.

## Summary

The demographic revolution constitutes a real challenge for society, but in particular for the older population and their helping professionals. The challenge for older adults is to maintain and, if possible, to increase their personal resources so as not to overwhelm societal resources with their needs. Thus, older adults should bear some responsibility for making sure that they maintain their health, maintain a healthy lifestyle, and are engaged in their families and communities. The challenge for helping professionals is to shift away from traditional stereotypes and ageism and suggest interventions that focus on wellness and older adults' reserve capacities rather than on illness, patients, and symptoms. Interventions should incorporate practicing positive aging and well-being strategies to increase autonomy, environmental mastery, and purpose in life, as well as a healthy lifestyle. A prime point of intervention is to identify the resources and facilitate the social network cooperation that will keep older adults socially and physically active and involved in their communities. It can be achieved together with the medical, physical, and mental health services given in health centers and primary care. Interventions should be adjusted to individual older adults' level of functioning, special needs, and motivation.

Although findings indicate that the majority of the older population has a resourceful interpersonal milieu, varied social networks, and positive well-being (81–83), there is also the risk of future disability and limitations in mobility, which can increase loneliness and reduce well-being. Increased loneliness and reduction in physical and mental health was also found in many older adults during the COVID-19 pandemic. It is recommended to recruit and train medical staff in health centers and primary care to incorporate strategies and tools for optimal functioning into their medical treatment approach. Crucially, the foundations for positive aging are laid down early in life by adopting a healthy lifestyle. It is important to develop healthy habits throughout the life cycle, which depends to a great extent on social and cultural contexts. Therefore, it is imperative to develop educational programs and interventions in the community to promote strategies for positive functioning and well-being. The challenge to present and future societies is to provide older persons with opportunities for self-realization, continued personal growth, and social engagements.

## Author Contributions

The author confirms being the sole contributor of this work and has approved it for publication.

## Conflict of Interest

The author declares that the research was conducted in the absence of any commercial or financial relationships that could be construed as a potential conflict of interest.
